# Use of the Sheathless Eaucath Is an Effective Strategy to Overcome Resistant Severe Radial Spasm

**DOI:** 10.1155/2023/2434516

**Published:** 2023-02-22

**Authors:** Andrew Borrie, Aditya Raina, Sarah Fairley, Anil Ranchord, Scott A. Harding

**Affiliations:** Wellington Hospital, Wellington, New Zealand

## Abstract

**Objectives:**

We aimed to assess the effectiveness of the sheathless Eaucath guiding catheter (SEGC) in overcoming severe spasm.

**Background:**

Radial spasm is a frequent challenge in transradial access (TRA) and can be difficult to manage.

**Methods:**

We performed a prospective observational study of 1000 consecutive patients undergoing coronary angiography with or without percutaneous coronary intervention. Patients with primary transfemoral access (TFA) or primary use of a sheathless guide catheter were excluded. Patients who developed angiographically confirmed severe spasm were treated with further sedation and vasodilators. If the conventional catheter would still not advance, it was exchanged for a SEGC. The primary endpoint was the successful passage of the SEGC through the radial with successful engagement of the coronary artery in patients with resistant severe spasm.

**Results:**

Primary TFA access was used in 58 (5.8%) and primary radial access with a SEGC in 44 (4.4%) patients. Of the remaining 898 patients, 888 (98.9%) had a radial sheath successfully inserted. Of these, 49 (5.5%) developed severe radial spasm with inability to advance the catheter. Following treatment with additional sedation and vasodilators, the severe spasm resolved in 5 (10.2%) patients. Passage of a SEGC was attempted in the remaining 44 patients with resistant severe spasm. Passage of the SEGC and engagement of coronary arteries were successful in all cases. There were no complications related to use of the SEGC.

**Conclusions:**

Our findings suggest that use of the SEGC for resistant severe spasm is highly effective, safe, and may reduce the need for conversion to TFA.

## 1. Introduction

Transradial access (TRA) for coronary angiography and percutaneous coronary intervention (PCI) has been shown to result in greater patient comfort, earlier mobilisation, reducing risk of vascular access complications, and reducing major bleeding when compared to transfemoral access (TFA) [1–5]. A recent patient level meta-analysis of multicentre randomised clinical trials comparing TRA versus TFA confirmed these findings and also demonstrated that TRA was associated with mortality reduction [6]. As a result, TRA is now recommended by current guidelines as the preferred access for coronary diagnostic and interventional procedures [7, 8].

Radial artery spasm remains a common challenge in transradial procedures. The occurrence of radial artery spasm has been variably reported to be between 4% and 20% [9, 10]. Severe radial spasm is less frequent occurring in 2.7–7% of patients [11, 12]. Severe radial spasm causes patient discomfort, difficulties with catheter manipulation, and is associated with high rates of crossover to femoral access [13, 14].

The sheathless Eaucath guide catheter (SEGC, Asahi Intecc Co., Japan) is a coronary guide catheter that has been developed to allow guide catheter introduction without use of an introducer sheath. The SEGC has a hydrophilic outer coating and a central tapered dilator that provides smooth transition between the guide wire and the guide catheter, design features that enhance trackability, and may aid passage through spastic segments. We have previously reported successful use of the SEGC to overcome severe spasm [15]. The aim of this study was to prospectively assess the effectiveness and safety of using the SEGC to overcome severe resistant radial spasm and complete the procedure via radial access.

## 2. Methods

This was a physician initiated, single centre prospective observational study. We evaluated outcomes in 1000 consecutive patients undergoing coronary angiography with or without PCI at our institution between December 2020 and October 2021. This study was approved by the local ethics committee.

**Figure 1:**
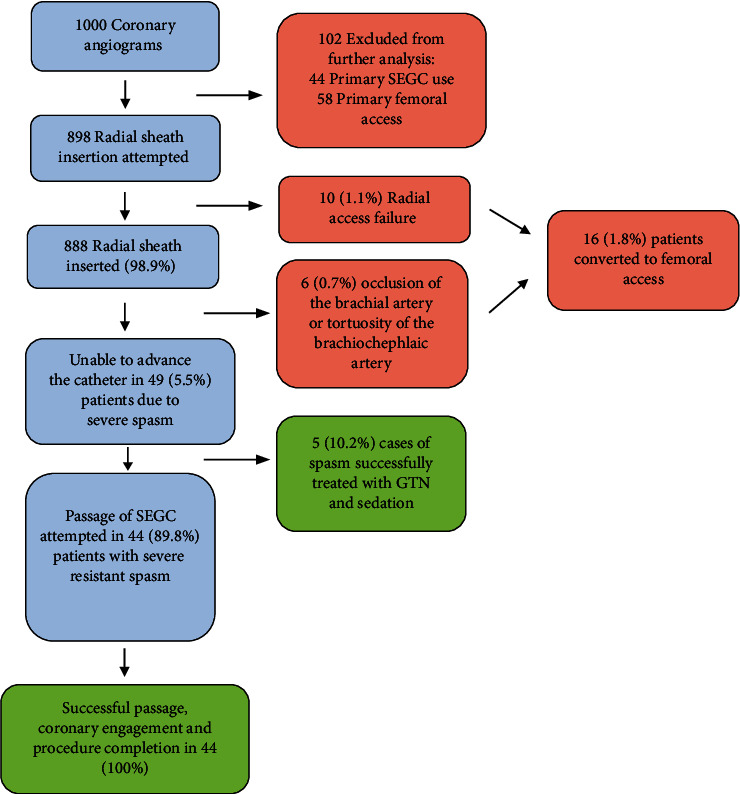
Study flow diagram.

Patients that had primary TFA or primary TRA using sheathless guide catheters were excluded from further analysis (Figure 1). Reasons for choice of the vascular access site were documented. If patients had dual TRA and TFA for bilateral injections only, the radial access was analysed. All patients were given sedation prior to radial access. Ultrasound guidance for radial artery puncture was used at the operator's discretion. Following radial sheath insertion, 200 mcg of intra-arterial glyceryl trinitrate (GTN) and 5000 IU of unfractionated heparin were given unless contraindicated. If a 0.35 guidewire was unable to be advanced through the radial artery, angiography was performed to confirm arterial anatomy. If tortuosity or spasm was demonstrated, the operator could change to a hydrophilic 0.35 wire or a 0.014 coronary wire at their preference.

Severe spasm was defined as severe local pain and discomfort during catheter movement along with an inability to advance the catheter. Angiography was performed in patients meeting the definition of severe radial artery spasm to confirm the diagnosis. If spasm was present, further sedation and GTN were given unless contraindicated. Intra-arterial verapamil was also administered in some cases at the operator's discretion. Resistant severe radial spasm was defined as the continued inability to advance the diagnostic or guiding catheter through the radial artery despite the use of additional sedation and vasodilators. In patients with resistant severe spasm, the existing catheter was exchanged for a SEGC. The SEGC was inserted through the existing radial sheath over a 0.035 guidewire. If the SEGC passed successfully, then radial angiography was recommended following completion of the procedure. If passage of the SEGC was unsuccessful, conversion to alternate access was considered.

The primary endpoint of this study was successful passage of a SEGC through the radial artery with successful engagement of the target coronary artery in patients with resistant severe spasm. Secondary outcomes included the need for conversion to TFA, vascular complications, angiographic success, and procedural success. Categorical variables are presented as numbers and percentages. Continuous variables are presented as mean ± standard deviation.

## 3. Results

Of the 1000 patients included, 942 (94.2%) had primary TRA and 58 (5.8%) had primary TFA. Reasons for primary TFA included previous coronary artery bypass grafting (CABG) in 27 (47%) patients, preservation of radial conduits in chronic kidney disease in 11 (19%) patients, previous problems with TRA in 8 (14%) patients, need for large bore access in 6 (10%) patients, and other reasons in 6 (10%) patients. A primary radial approach with a sheathless guide catheter was undertaken in 44 (4.4%) patients. Sheathless guide catheters were chosen to facilitate the use of larger size guide catheters or in patients with previous significant spasm. None of the patients with a primary sheathless approach developed severe spasm during the study procedure. Patients with primary TFA and primary TRA using a sheathless guide catheter were excluded from further analysis.

Patient demographics and clinical characteristics of the remaining 898 patients who had had primary radial access using an introducer sheath are described in Table 1. The mean age was 65 ± 11 years, 71% were male, and 64% had an acute coronary syndrome.

Radial sheath insertion was successful in 888 (98.9%) (Figure 1). Radial sheath sizes were as follows: 24 (2.7%) 5 F, 859 (96.7%) 6 F, and 5 (0.6%) 7 F. The procedures performed in this group were coronary angiography alone in 389 (43.8%), coronary angiography and PCI in 402 (45.3%), and PCI alone in 97 (10.9%). Crossover to femoral access was required in 6 (0.7%) patients with successful radial sheath insertion due to brachial occlusion or brachiocephalic artery tortuosity.

**Figure 2:**
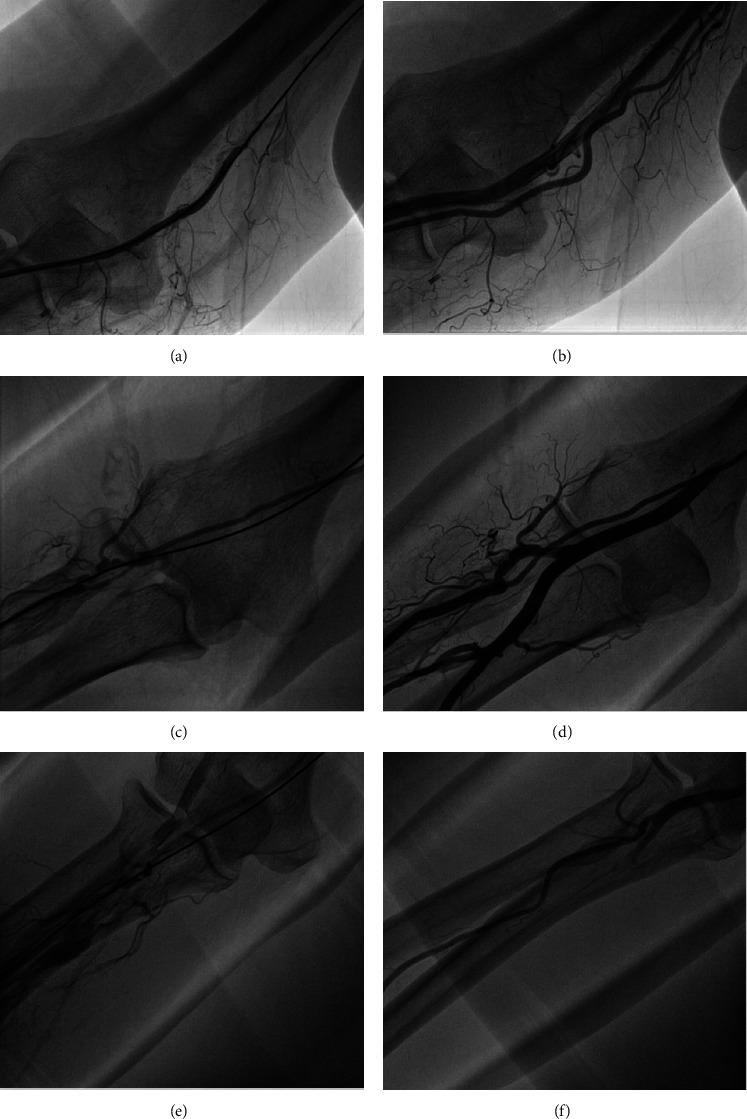
(a) Radial angiogram demonstrating occlusive radial spasm. (b) Radial angiogram following completion of the procedure with a sheathless Eaucath guiding catheter (SEGC) demonstrating a high origin of the radial artery with resolution of previous spasm. (c) Radial angiogram demonstrating tortuosity and severe spasm of the radial artery. (d) Radial angiogram following completion of the procedure with a SEGC demonstrating moderate tortuosity with resolution of previous radial spasm. (e) Radial angiogram demonstrating radial spasm and perforation. (f) Radial angiogram following completion of the procedure with a SEGC demonstrating resolution of the radial spasm and perforation.

Severe spasm with failure to advance the catheter occurred in 49 patients (5.5%). All had spasm angiographically confirmed. Further findings on radial angiography in the patients with severe spasm included a high origin of the radial artery in 12 (24.5%) patients, tortuosity in 3 patients (6.1%), the partial or full radial loop in 2 patients (4.1%), and perforation or dissection in 3 patients (6.1%) (Figure 2). Of those with severe spasm, 5 (10.2%) were able to be managed successfully with further vasodilators and sedation leaving 44 patients (89.8%) who met the definition of resistant severe spasm in whom passage of a SEGC was attempted. The sizes of the SEGC used were 6.5 F in 43 (98%) patients and 7.5 F in 1 (2%) patient. The primary outcome of the study, successful passage of a SEGC through the radial artery with successful engagement of the target coronary artery, was achieved in all 44 patients with resistant severe spasm. All procedures in patients with resistant spasm (coronary angiography in 13 (29.5%) and PCI in 31 (70.5%)) were completed successfully. Of these 44 patients, none had clinically evident complications and 30 (68%) underwent repeat radial angiography upon completion of the procedure with no radiographic evidence of complications. The two perforations that had been documented prior to the passage of the SEGC had sealed at the time of completion of the PCI.

## 4. Discussion

To the best of our knowledge, this is the first study to prospectively assess use of a sheathless guide catheter to overcome severe radial spasm. Our main finding was that use of the SEGC was highly effective and safe in the management of resistant severe spasm with all cases being completed successfully without the need for crossover to TFA or complications. These findings suggest that the SEGC may be a simple and safe strategy for overcoming severe radial spasm.

In our study, there was a high rate of successful radial access, with rates of spasm being similar to those reported in previous large studies [2, 12]. We found anomalous radial anatomy was frequently present (34.7%) in those with severe resistant spasm. Anomalous radial artery anatomy has been reported previously to be present in 14% of all patients undergoing transradial procedures [16]. The high frequency of anomalous radial anatomy in patients with resistant severe spasm is expected as spasm and TRA failures have previously been shown to be higher in those with anomalous anatomy [16].

The SEGC performed well in patients with resistant severe spasm across a range of radial artery anatomical variants. In this study, no patient required conversion to TFA due to radial artery spasm and procedural success was achieved in all patients with resistant severe spasm. The 2 perforations that occurred were the result of attempts to pass a conventional guide catheter through an area of severe spasm. The presence of these perforations was not suspected clinically but was noted when angiography was performed to confirm the presence of spasm and define the anatomy prior to the introduction of a sheathless Eaucath. In both cases, the sheathless Eaucath passed easily and the case was completed successfully. Radial angiography performed on removal of the sheathless Eaucath demonstrated that the perforation had sealed in both cases. Although perforation can necessitate conversion to transfemoral access, several other reports have demonstrated the safety of continuing the procedure with the use of a long sheath, guiding catheter, or peripheral balloon to seal the perforation [17–19]. Furthermore, none of the patients in these series experienced a long-term vascular complication.

There are several design features of the SEGC that may contribute to its success in the setting of spasm. A tapered central dilator is inserted through the sheathless Eaucath to aid introduction over a 0.035” angiographic guide wire. The tapered shape of the dilator, absence of a gap between the tip of the dilator, and the 0.035” guide wire along with the smooth transition between the dilator and the sheathless Eaucath are all design features likely to facilitate entry of the SEGC into the spastic segment. The sheathless Eaucath also has a hydrophilic coating along its length designed to reduce friction between the Eaucath and the vessel wall which is likely to enhance trackability through spastic or tortuous vessels. This assumption is supported by previous observations that hydrophilic coatings on introducer sheaths of reduced rates of radial spasm are reduced by the use of hydrophilic coatings on introducer sheaths [20, 21] and that catheters with hydrophilic coatings are more likely to pass across vascular tortuousity [9, 22].

The use of sedation, intra-arterial vasodilators, hydrophilic radial sheaths, and smaller catheters in addition to minimizing catheter manipulation are all important in reducing radial artery spasm [23]. Despite utilization of these techniques, spasm can occur even when experienced operators are performing procedures. As demonstrated in the current study, unexpected spasm is often associated with the presence of vascular abnormalities. When resistance to catheter passage is encountered, we strongly advise use of angiography to determine if spasm is the problem and to identify if any anatomic variants are present. We believe that angiography is mandatory before trying to use a SEGC to overcome spasm. In all cases in this study, the SEGC passed easily. It is important to avoid pushing the catheter if significant resistance is encountered as this could lead to vascular injury. We and others have previously reported that balloon-assisted tracking is also an effective method to advance catheters [15] in the setting of spasm. It is also possible that other sheathless guide catheters or sheathless access systems such as the Cordis RAILWAY system may be effective in overcoming spasm.

## 5. Limitations

The main limitation of this study is that it is an observational study from a single centre. This study was performed in a high volume tertiary centre with experienced transradial operators, familiar with the use of the SEGC, and this should be taken into account in interpreting the study results. Although a large number of patients were entered into our study, the number of patients with severe resistant spasm in which the SEGC was used was relatively small. Thus, infrequent complications related to this technique may not have been detected. In addition, follow-up was limited to the time of discharge, so we cannot exclude the possibility of late complications. Previous studies have demonstrated that the vast majority of complications related to radial access occur in hospital.

## 6. Conclusion

Radial artery spasm is one of the major procedural challenges associated with the transradial approach. Conventional strategies currently used are at times insufficient to overcome severe spasm and may result in crossover to the femoral access or procedure abandonment. The results of this study suggest that use of the SEGC is a simple, highly effective, and safe method for overcoming resistant severe radial spasm avoiding the need for crossover to TFA.

## Figures and Tables

**Table 1:** Patient demographic and clinical data.

Variables	*n* = 898
Age (years)	65 ± 11
Male sex (%)	71%
Median BMI (kg/m^2^)	24.5±
Hypertension (%)	48%
Hyperlipidaemia (%)	28%
Diabetes mellitus (%)	21%
Current smokers (%)	13%
Previous MI (%)	21%
Previous PCI (%)	25%
Previous CABG (%)	6%
Acute coronary syndrome (%)	64%

BMI, body mass index; CABG, coronary artery bypass grafting; MI, myocardial infarction; PCI, percutaneous coronary intervention.

## Data Availability

The data used to support the findings of this study are restricted to protect patient privacy. Data are available from the corresponding author for researchers who meet the criteria for access to confidential data.
